# *In-vivo* Investigations of Hydroxyapatite/Co-polymeric Composites Coated Titanium Plate for Bone Regeneration

**DOI:** 10.3389/fcell.2020.631107

**Published:** 2021-02-18

**Authors:** Weilong Diwu, Xin Dong, Omaima Nasif, Sulaiman Ali Alharbi, Jian Zhao, Wei Li

**Affiliations:** ^1^Department of Orthopedics, The First Affiliated Hospital of Air Force Military Medical University, Xi'an, China; ^2^Department of Orthopedics, The Second Affiliated Hospital of Air Force Military Medical University, Xi'an, China; ^3^Department of Physiology, College of Medicine and King Khalid University Hospital, King Saud University, Riyadh, Saudi Arabia; ^4^Department of Botany and Microbiology, College of Science, King Saud University, Riyadh, Saudi Arabia

**Keywords:** polymeric composite, hydroxyapatite, osteoporosis, titanium plate, *in-vivo*

## Abstract

A perfect mimic of human bone is very difficult. Still, the latest advancement in biomaterials makes it possible to design composite materials with morphologies merely the same as that of bone tissues. In the present work is the fabrication of selenium substituted Hydroxyapatite (HAP-Se) covered by lactic acid (LA)—Polyethylene glycol (PEG)—Aspartic acid (AS) composite with the loading of vincristine sulfate (VCR) drug (HAP-Se/LA-PEG-AS/VCR) for twin purposes of bone regenerations. The HAP-Se/LA-PEG-AS/VCR composite coated on titanium implant through electrophoretic deposition (EPD). The prepared composite characterized using FTIR, XRD techniques to rely on the composites' chemical nature and crystalline status. The morphology of the composite and the titanium plate with the composite coating was investigated by utilizing SEM, TEM instrument techniques, and it reveals the composite has porous morphology. The drug (VCR) load in HAP-Se/LA-PEG-AS and releasing nature were investigated through UV-Visible spectroscopy at the wavelength of 295 nm. *In vitro* study of SBF treatment shows excellent biocompatibility to form the HAP crystals. The viability against MG63 and toxicity against Saos- 2 cells have expressed the more exceptional biocompatibility in bone cells and toxicity with the cancer cells of prepared composites. The *in-vivo* study emphasizes prepared biomaterial suitable for implantation and helps accelerate bone regeneration on osteoporosis and osteosarcoma affected hard tissue.

## Introduction

Osteosarcoma is a cancer that frequently occurred on bone, caused harmful essential bone cancer with a high fatality rate, both in children and young people (Khanna et al., 2004; Khan et al., 2019). Since the cancer drug-loaded bone regeneration materials are much attention in tissue engineering applications. The sarcoma is mainly affected in the long bones, particularly the legs and arms, but it can start any bone. The sugary is the primary treatment for the sarcoma affected a bone repair, and after the surgery, may cancer have left in the sarcoma affected bone surrounding tissues. Vincristine (VCR), a chemotherapeutic agent. It is used as an antitumor drug; its relics a useful and broadly used anticancer drug, predominantly for childhood and adult hematologic malignancies and solid cancer with sarcomas. VCR ties for all time to microtubules and shaft protein in the S period of the cell cycle and meddles with the course of action of the mitotic axle, along these lines striking tumor cells in metaphase (Goto et al., 2007). This accomplishment might direct to the VCR's resulting cell death besides the cell proliferation rate. The bone uniting by utilizing whichever autografts or allografts has been the “best quality level” therapy for patients experiencing essential bone imperfections; however, there are disadvantages, for example, difficulties incomplete joining of bone tissue to the implantation (Hao et al., 2018). Tissue engineering signifies a united perspective to rehabilitate or regenerate affected tissue by connecting biology and engineering (Perikamana et al., 2015; Chahal et al., 2019).

Titanium and its alloys developed for enduring implant utilizing for their favorable excellent mechanical properties, corrosion resistance, as well as their low dangers, biocompatibility, and such as elevated potency, stability, and less heaviness (Teshima et al., 2012; Sumathra and Rajan, 2019). However, they need particular surface treatment to defeat problems such as less bio-functionality, toxicity, or immunogenicity (Geuli et al., 2017). Metal bone implants are subjected to physical or chemical modification to improve wear resistance, osteointegration, and biomechanical compatibility nature. Different types of surface alteration methods are available such as modified by sandblasting and acid engraving for surface coarseness and coating of bioactive materials (Geetha et al., 2009; El Hadad et al., 2014; Fu et al., 2016; Harun et al., 2018). The electrophoretic deposition method (EPD) appears to be more suitable to create homogeneous and dense ceramic polymer composite coating at compact outlay for medical significance (Sun et al., 2009; Gebhardt et al., 2012). Electrophoretic statement (EPD) is an adaptable and least expensive method for the deposition of the metallic substrate through pulse current deposition. The EPD deposition, the continuous, homogenous layer with uniform grain size and the complete coating, was obtained, and it has improved mechanical and substance properties (Sumathra et al., 2018; Mehnath et al., 2020).

Specific material nature impacts the improvement of macroscopically various bone structures *in-vivo* with personalized shapes, mechanical strength, and spatial deliveries. Many bone substitute materials proposed to trade the requirement for autologous or allogeneic bone have been assessed in the most recent 20 years. Commonly, the researcher comprises of either bioactive glasses, bioactive ceramics, synthetic and natural polymers, and composites (Kretlow and Mikos, 2007; Rahman et al., 2015). The ideal fundamental reason in the tissue regenerations is that the materials will be resorbed and supplanted over the long run by, and on top of, the body's own recently recovered organic tissue (Rahman et al., 2015). Bioceramic materials facilitate give with appropriate physicochemical and biochemical cues that have gathered considerable concern for both *ex-vivo* and *in-vivo* tissue rejuvenation methodologies (Naderi et al., 2011). The first inorganic module of human hard tissue, hydroxyapatite (HAP), has fascinated widespread significance in biomedical and scientific relevance, particularly in bone regenerations (Bose Susmita et al., 2010). The HAP reveals a superior suitable act because of the nanometer range's more specific surface area, with the supportive enhancement of various biological goods to interact bone cell and tissues (Zhou et al., 2019; Sumathra et al., 2020). Besides, HAP coating on Ti plate can reduce the implant fiction time, improve attachment amid implants and host tissues, and generate consistent bone augmentation close to the bone-implant boundary (Ghosh et al., 2019). Even though HAP is an osteoconductive material, it lacks osteoconductivity to enhance bone growth. Since the mineral substituted and polymer reinforced composite overcome the issues implanted related contaminations are gotten from bacterial infections. The biofilm development at the implantation site and the restraint of bacterial bond are considered the essential advance in post-implanted diseases (Zilberman and Elsner, 2008). The Selenium (Se) nanoparticles are newly rising as capable antibacterial and anticancer agents due to their high bioavailability, considerably lesser toxicity than selenium compounds. It represents that Se could be appropriate as a curative applicant to bacterial infectious disease (Xia et al., 2018).

Besides, to overcome the reduced bone bonding activity, soft nature of the materials and improve its bioactivity to achieve an ideal bone repair requirement, more attention is paid to fabricate polymer reinforced HAP/Se composite (Yanhua et al., 2016; Zhou et al., 2020). The artificial bioabsorbable polymers can encourage isolated cells to redevelop tissues. Since here Lactic acid-Poly ethylene glycol-Aspartic acid copolymer used as scaffold material, the finding of PEG interaction with the cell membrane to give cell fusion, becomes a primary platform for PEG in biomedical, tissue engineering for satisfactory development of the bone-implant materials (Zhou et al., 2020). Based on these, a biomimetic bone-like composite, made of the anticancer drug (VCR) loaded HAP/Se/LA-PEG-AS polymer composite, has been deposited by an electrochemical method on a titanium plate. This work innovative is to the synthesized composite coated surface-treated Ti implant might be acting as better bone regeneration ability, antibacterial activity, and anticancer activity after the post-surgical implantation. The VCR loaded HAP/Se/LA-PEG-AS polymer composite will serve as a potential implant to overcome the drawbacks of the exciting bone implant materials.

## Materials and Methods

### Materials

Sodium alginate (SA) was bought from SD Fine Chemical Ltd, China. Polyethylene glycol (PEG6000), L-Aspartic acid (C_4_H_7_NO_4_), Calcium chloride dihydrate (CaCl_2_.2H_2_O), Diammonium phosphate (N_2_H_9_PO_4_) obtained from SRL chemicals, China. Lactic acid (C_3_H_6_O_3_) bought from Himedia chemicals, China. Selenium dioxide and L-ascorbic acid used as received. Ammonium fluoride (NH_4_F) and Vincristine sulfate drug (Chemotherapeutic drug) were acquired from Sigma-Aldrich, China. All analytical grade types of chemicals are used as such. Double Distilled (DD) water was used all over the experimental reactions.

### Preparation of Selenium Nanoparticles

0.035 g of selenium dioxide was liquefied in 5 mL of distilled water and placed in a magnetic agitator. After 10 min, to the solution, 0.105 g of sodium alginate dispersed in 5 mL of water was added in a dropwise manner. In the colorless solution, 0.035 g of L-ascorbic acid was added slowly. During the addition, the color of the solution gradually changes from colorless to the yellow solution, and finally to brick red color indicates the formation of selenium nanoparticle. And it was allowed in a stirrer for 30 min at room temperature (RT) (25°C). Then it was washed, filtered, and dried in an oven at 60°C.

### Preparation of Se Impregnated HAP

Calcium chloride dihydrate of (0.6615 g) was liquefied in 10 ml of DD water, and selenium nanoparticles in the liquid phase of 0.05 moles were added to a colorless solution of Calcium chloride dihydrate by placing it in a magnetic stirrer. The 0.3 moles of ammonium phosphate solution (0.396 g) in 10 ml of DD water were added dropwise to the above mixture. Brick red colored colloidal solution of HAP-Se obtained, and the pH adjusted to 9.0–10.0 by using aqueous ammonia, and it stirred for 6 h at 25°C. Further, the solution was ultra-sonicated with 30 amplitude under 3-s pulse on and 2-s pulse off condition for 30 min at Room Temperature (25°C). Later the precipitate was washed with DD water repeatedly, filtered, and dried to get a fine powder, and it is sintered for 6 h in a muffle furnace at 600°C. The above procedure followed for the preparation of HAP particle without Selenium solution.

### Preparation of LA/PEG/AS Co-polymer

1.2 g of polyethylene glycol (PEG) dissolved in 10 mL of distilled water in a round bottom flask. Then 0.013 g of aspartic acid in 10 mL DD water was added by keeping it in an oil bath at 120° under stirring conditions. A few min later, the lactic acid solution of 7.4 μl was added and allowed to esterify for 3 h by condensation method. The water condenser is placed over the reaction flask to maintain the constant heat. Finally, the solid-phase copolymer was obtained and further dehydrated using microwave irradiation.

### Preparation of HAP/Se/Co-polymer Composite

0.2 g of prepared HAP was taken in 10 mL of water and sonicated, and placed in a stirrer. To this solution, 20 wt% (0.04 g) of prepared LA-PEG-AS polymer dissolved in 4 mL of water was mixed gently and stirred for 30 min. It was ultra-sonicated under probe sonication with 30 amplitude under 3-s pulse on and 2-s pulse off condition for 30 min at 25°C. The dried HAP/Se/polymer composite was collected, and then it autoclaved at 180°C.

### Preparation of Drug Loaded Composite

One gram of HAP/Se/Polymer composite was dissolved in distilled water and stirred in a magnetic stirrer. The 5 wt% of vincristine drugs (50 mg) were added and stirred for a 1 h at 25°C. Then it was lyophilized to get dried drug-loaded composite for further investigations.

### Surface-Modification of Titanium Plate by Anodization

The titanium foils with a purity of 99.99% were used in this work, which was obtained from Sigma Aldrich. The process of anodization was carried out at 25°C with magnetic agitation. The anodization process etches a titanium plate. The titanium plate serves as the anode, and the platinum electrode serves as the cathode. These electrodes are kept in a prepared solution and connected to the DC power supply. The voltages for the anodizing process were kept constant throughout the whole process. In the first 5–10 s of the anodization, the currents were observed to decrease drastically and then remained stable, and it continued up to 1 h at 25°C. During the anodization process, the titanium oxide layer's color normally changed from purple to blue to light red. The final step of pretreatment was cleaning the Ti plate with acetone and deionized water. The substrate then dried in air at room temperature. The plate was washed with water and annealed at 600°C for 4 h.

### Synthesis of Drug Loaded Nanocomposite Coated Titanium Implant

The 20 mg (0.02 g) of HAP-Se/LA-PEG-AS/VCR composite dispersed in the 20 mL of isopropyl alcohol contain a beaker through homogenization. Then, it was placed in a magnetic stirrer. Surface treated titanium plate inserted. It acted as cathode and Platinum electrode acting as anode both in the immersed in the sample solution and the electrodes connected to the DC power supply. The prepared composite gets deposited on the surface-treated Ti plate by applying 20V for 1 h at room temperature (25°C) dried at room temperature, and stored for further studies.

### Physicochemical Characterizations

#### Fourier Transforms Infrared Spectroscopy (FTIR) Analysis

The HAP, Se, HAP-Se, LA-PEG-AS, HAP-Se/LA-PEG-AS, and HAP-Se/LA-PEG-AS/VCR composites tried by a Bruker Tensor 27 Series FTIR spectrometer and 16 sweeps for each example taken in the area of 400–4,000 cm^−1^ with 2 cm^−1^ goals. The pellets were made for the FTIR test by smashing 0.2 g of test powder along with 1 g KBr and squeezing them into a straightforward circle.

#### X-ray Diffraction Determination

The X-ray diffraction (XRD) characterization was done to analyze the phase composition and to precisely obtained the crystallinity of prepared HAP, Se, HAP-Se, LA-PEG-AS, HAP-Se/LA-PEG-AS, and HAP-Se/LA-PEG-AS/VCR composites. The XRD examination achieved in a Bruker D8 Advance Diffractometer with monochromatic Cu Kα source worked at 40 kV and 30 mA. A quickening voltage of 30 kV and a current of 15 mA was applied. The working scope of this test was over the 2θ scope of 10–60° in sync check mode with a stage size of 0.02°and a sweeping pace of 0.02°/min.

#### Scanning Electron Microscopy (SEM)

The morphology of the HAP, Se, HAP-Se, LA-PEG-AS, and HAP-Se/LA-PEG-AS/VCR mixtures were inspected by SEM (VEGA3 TESCAN) by operating it at an extent voltage of 10 Kv.

#### Transmission Electron Microscopy

The outside of blended HAP-Se/LA-PEG-AS composites and VCR stacked HAP-Se/LA-PEG-AS composites dictated by transmission electron microscopy (HR-TEM, TECNAI F30). For the test arrangement of the HR-TEM investigation, the incorporated nanoparticles and their composites were scattered in ethanol by ultra-sonication up to 15 min. A short time later, these then stacked on a carbon-covered copper work.

#### Contact Angle Analysis

Wettability of the HAP-Se and HAP-Se/LA-PEG-AS/VCR subjectively analyzed utilizing the estimating water contact point (WCA) utilizing a contact edge goniometer (Model OCA EC15 from Data Physics, GmbH, Germany) outfitted with inner picture examination programming. Refined water (2 μL) dropped on the outside of dry sample platforms at room temperature, and the wetting process was recorded utilizing a rapidly advanced camera.

#### Loading Capacity(LC) of HAP-Se/LA-PEG-AS

UV-Visible spectroscopy was utilized to study the loading capacity of the HAP-Se/LA-PEG-AS composites. Initially, the 10 mg of HAP-Se/LA-PEG-AS/VCR composite was mixed 3 ml of acetone, and the composite was stirred for 3 h. Afterward, the solution was centrifuged, the supernatant solution was measured in UV-Visible spectroscopy at λ_max_ value of 295 nm (Kolmas et al., 2014). The following equation calculated the loading capacity.

$$\begin{array}{l} LC\left(\%\right)=\frac{Total\;amount\;of\;VCR-Free\;amount\;of\;VCR}{Total\;amount\;of\;VCR}\times 100 \end{array}$$

#### In-vitro Release Studies

The *in-vitro* release of VCR from HAP-Se/LA-PEG-AS/VCR composite was determined through a dialysis membrane procedure utilizing a PBS arrangement working at pH 7.4, and the process was followed by the previous literature (Djordjevic et al., 2002; Kolmas et al., 2014). Test sample preparation incorporated the sealing of 50 mg of HAP-Se/LA-PEG-AS/VCR composite into independent dialysis bags with the MWCO (12000Da). At that point, 10 mL of the PBS solution contain VCR loaded composites mixed under 100 rpm at 25°C. The supernatant solution was collected at different day intervals by measuring the concentration of VCR solution at λ_max_ value of 295 nm in a UV-Spectroscopy and replenishing it among an identical quantity of new PBS medium.

The following formulae used to calculate the % of drug release

$$\begin{array}{l} Drug\;release\;\left(\%\right)\;=\frac{AR}{AC}\times 100 \end{array}$$

AR is the Absorbance of VCR discharged from the composite, and AC is the sum amount of VCR loaded in the composite.

### Biological Analysis

#### Strain and Culture Condition

Methicillin-resistant Staphylococcus aureus (MRSA) was gotten from the American Type Culture Collection (ATCC) and preserved in Tryptone soy Broth (TSB) (HiMedia, China) at −80°C. For each experimentation, 10 μL of bacterial stock culture was used to immunize TSB and hatched at 32°C for 24 h. For biofilm assay, Brain heart infusion Broth (BHIB) is used to inspire biofilm formation (Premaratne et al., 1991; Kannan and Alice, 2017).

#### MIC Determination

To research the antibacterial action of HAP-Se/LA-PEG-AS and HAP-Se/LA-PEG-AS/VCR, MIC of different mixes of scatterings against MRSA determined using the microtitre broth dilution technique rendering to the strategies of the Clinical and Laboratory Standards Institute. The particles were sonicated before the antibacterial examination to confirm the homogenous dispersal of the particles. All the dispersants were diluted in a 96-well microtitre plate (MTP) holding Mueller–Hinton broth MHB to attain final concentrations ranging from 1 to 500 μg/l.5 μL Bacterial suspensions with 108CFU/mL included into each well barring for the sterility control MHB spaces stock. The tops were fixed, and the titer plates were hatched at 37°C for 24 h and assessed. The bacterial development was surveyed by estimating the optical thickness of the way of life at 600 nm, utilizing a Spectramax microtitre plate reader (Molecular Devices, Sunnyvale, USA).

#### Assessment of Biofilm Biomass

The impact of polymer buildings on MRSA biofilm development was examined in 48-well MTP, as depicted by Kannan and Alice (2017) with a slight alteration. Briefly, polymer complex included in sub-MIC (½ MIC) to MWB containing a bacterial suspension of 10^8^ CFU/ml and incubated at 32°C for 48 h. After incubation, the OD_600was_ recorded spectrophotometrically to assess drug and drug-loaded complex impacts on *MRSA* growth. Thus, planktonic cells from the wells were evacuated and were dissolved 3-fold with sterile PBS, and the followed sessile cells recolored with 0.4% precious crystal violet (CV) arrangement. After 5 min incubation, the stain evacuated, and the wells washed with sterile refined water and excess water blotted. In the wake of drying, 20% cold, acidic acid was used to disintegrate the recolored biofilms for 30 min, and the biofilm biomass was evaluated spectrophotometrically at OD 570 nm. The level of biofilm hindrance is determined by the strategy for Kannan and Alice (2017).

#### Prevention of Hemolysis

RBC's lysis was quantified by incubating the treatment combinations with culture filtrates with an equal volume of 2% sheep red blood cells (RBCs) in phosphate-buffered saline (pH 7.4) at 37°C for 2 h. The reaction combination was centrifuged at 8,000 rpm for 5 min at 4°C OD _530_ was recorded with the supernatant (Jordan, 2002).

#### Inhibition of Slime Production With Congo Red Agar (CRA)

Colony morphologies and phenotypic changes researched utilizing CRA, as recently portrayed. The CRA was made out of 37 g/L of cerebrum heart implantation stock (Himedia), 36 g/L of sucrose,15 g/L of agar (BD Biosciences, Franklin Lakes, NJ, USA), and 0.8 g/L of Congo red (Sigma, St. Louis, MO, USA). MRSA cells on CRA were brooded with and without HAP-Se-HSPD for 24 h at 37°C before taking pictures.

#### Cell Viability Test Assays

The osteoblast-like MG63 cells were secured from the Cell Bank of Type Culture Collection of the Chinese Academy of Sciences, China. The cells were kept up at 37°C in a CO2 hatchery (with a humidifier) in Dulbecco's changed Eagle's Medium (DMEM) enhanced with 10% Fetal Bovine Serum and 1% penicillin/streptomycin. Collecting of the cells played out like clockwork utilizing a trypsin/EDTA arrangement. The composites against human osteoblasts like cells MG63 cells checked using a corresponding MTT (3-(4, 5-dimethyl thiazol-2-yl)- 2,5-diphenyl tetrazolium bromide) test. MG63 cells were seeded in a 24-well plate at a thickness of 4 × 104 cells/well. They were then co-refined with HAP-Se/LA-PEG-AS and HAP-Se/LA-PEG-AS/VCR with MG63 cells as a control cell line for the composites. Cell reasonability of osteoblasts, for example, MG63 cells, surveyed utilizing the MTT measure. After brooded for 1, 3, and 7 days, test arrangements were isolated, and 100 μL MTT arrangement (5 mg/mL) added to 1 mL of the way of life medium in each well plate. The hatched example cells were trailed by brooding at 37°C for 4 h. At that point, 1 mL of dimethyl sulfoxide (DMSO) included the plate, and the supernatant medium was gathered by centrifugation. The OD esteems supernatant arrangement recorded at a frequency of 490 nm utilizing a microplate peruser.

#### Cytotoxicity

Cell separation was examined utilizing Saos-2 cells, which were bought from the Cell Bank of Type Culture Collection of the Chinese Academy of Sciences, China. The Saos-2 cells were seeded in 24-well plates containing Dulbecco's altered falcon medium (DMEM) enhanced with 10% FBS and penicillin 100 U/mL-streptomycin (100 U mL-1) (Gibco, Grand Island, CA, USA) and developed for 24 h. The cells brooded at 37°C (RT) in CO2, and they saw utilizing MTT examine methods. HAP-Se/LA-PEG-AS, HAP-Se/LA-PEG-AS/VCR, the composites tried on cells for 1, 3, and 7 days. OD esteems recorded at λ_max_ of 490 nm in a microplate peruser. The accompanying equation used to figure the cytotoxicity of composite:

$$\begin{array}{l} \text{Cytotoxicity}\left(\%\right)=TestSample/Control\times 100 \end{array}$$

#### Fibroblast L929 Cell Viability

Mouse connective tissue fibroblast (L929) cell used to evaluate suitability and expansion for the readied composite biomaterial. The HAP-Se/LA-PEG-AS and HAP-Se/LA-PEG-AS/VCR with the grouping of 10 μg/ml grouping were inundated in 70% ethanol for 5 min cleansing, followed with dissolvable supplanted by deionized water. At that point, the composites set on a 24-well polystyrene plate and culture medium enhanced to each well before cell seeding. Cells were allowed to essential join for 5 h. For expansion testing, cells seeded onto every one of the materials, and societies collected after 1, 3, and 7 days. The joined or multiplied cells were then evaluated by the 3-(4, 5-dimethyl thiazolyl-2)- 2, 5-diphenyltetrazolium bromide (MTT) test. MTT arrangement 0.5 mg/ml in Dulbecco's changed bird medium (DMEM) (without phenol red, channel cleaned) was added to each culture well. After hatching for 5 h, the MTT response medium was expelled, and 900 μl of dimethyl sulfoxide and 100 μl of glycine cradle (pH-10.5) were enhanced. A spectrophotometer found the optical densities at the frequency of 570 nm.

#### Rat Surgery

For *in-vivo* animal studies, the animals were allowed to become acclimatize for a minimum of 2 weeks before testing. All the animal research was approved by the animal ethical committee of the second affiliated hospital (Tangdu Hospital), Air Force Military Medical University Approved No. SCXK (army) 2019-214. The HAP-Se/LA-PEG-AS/VCR coated titanium plates were implanted in six Wistar male rats (*n* = 6) with a weight of 200–250 g. Rats were separately anesthetized via intraperitoneal injection with the combination of ketamine (20 mg/kg) and xylazine (2 mg/kg) by exposure to 20% (v/v) isoflurane and propylene glycol. A bone imperfection of the tibia in the size-10 × 10 × 1 mm on the rats was induced using an electrical drill (supreme micrometer) and a sterile bur under irrigation with sterile normal saline. Titanium plates (10 × 10 × 1 mm) were totally inserted inside the tibia bone on the bony defect. The surgical process involved removing the hair over the outer region of the tibia via shaving and cleaning. The skin was sanitized with 10% betadine and stitched with sutures. The animals were provided with appropriate prophylactic anti-infection agents, and they were kept in large enclosures to facilitate ambulation during the final stage. All animals were examined every week for any sign of infection or discomfort on the tibia for the last period. All the implants were retrieved after the corresponding stage (Murugan et al., 2018).

#### Histopathology Analysis

The animals were sacrificed by exposure to CO_2_; every rat's tibia was removed and sectioned for histological examinations. Subsequently, the implants were harvested and evaluated histologically after 4 weeks (*n* = 6) implantation. The light microscopy was used for analysis, and the sample was fixed in 10% paraformaldehyde solution at paraffin, sectioned, and then stained with Masson's trichrome and hematoxylin & eosin (H&E). The stained sections of each test sample were then examined using light microscopy (Murugan et al., 2018).

### Statistical Analysis

The experiments repeated a minimum of three times expressed as a mean ± standard deviation using ANOVA. In all tests, statistical significance was set at *P < 0.05.

## Results

### FTIR Spectroscopy Analysis

**Figure 1:**
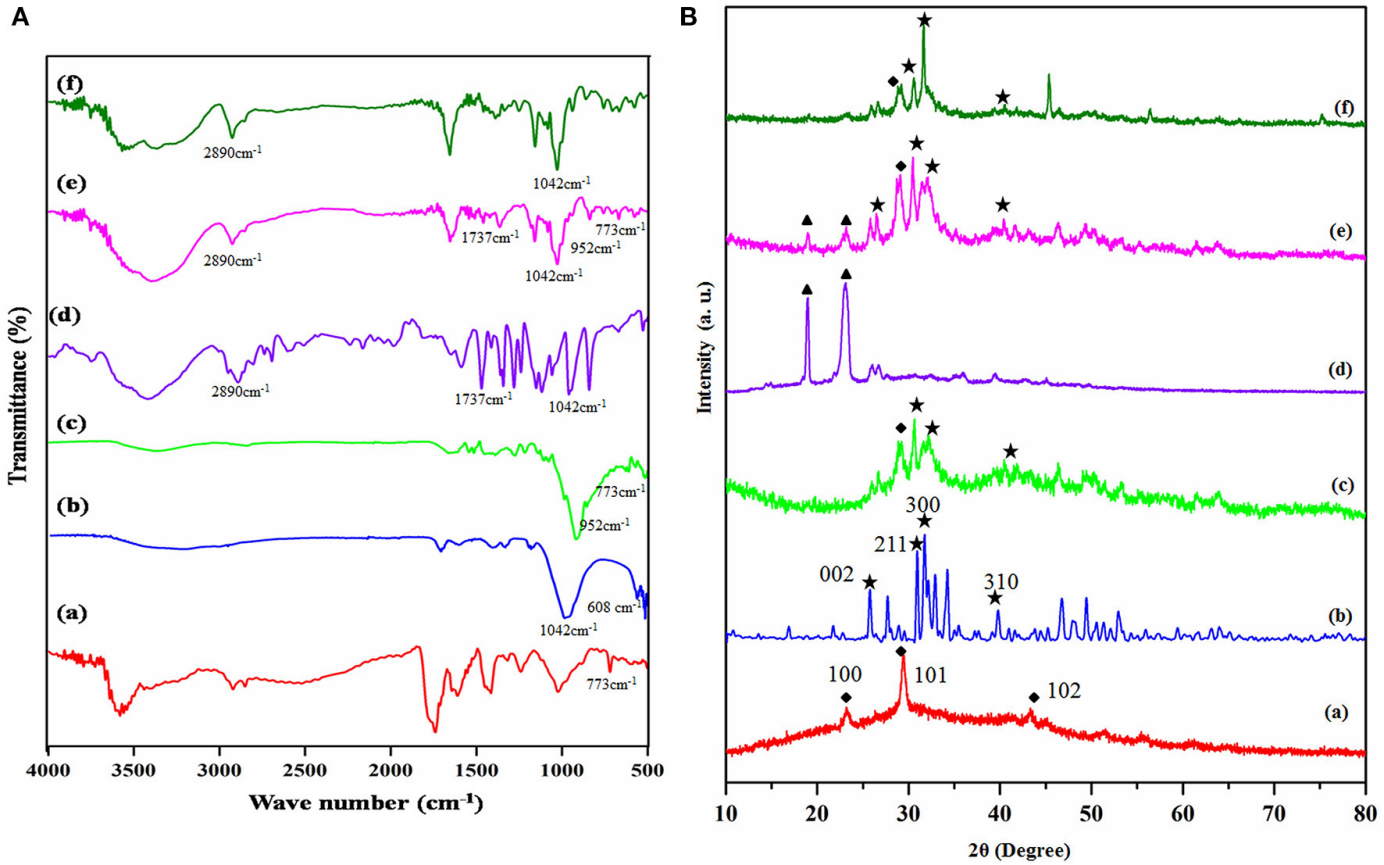
**(A)** FT-IR spectra of (a) Se-NPS, (b) HAP, (c) HAP-Se, (d) LA-PEG-AS, (e) HAP-Se/LA-PEG-AS, (f) HAP-SE/LA-PEG-AS/VCR; **(B)** XRD spectra of (a) Se-NPS, (b) HAP, (c) HAP-Se, (d) LA-PEG-AS, (e) HAP-Se/LA-PEG-AS, (f) HAP-SE/LA-PEG-AS/VCR.

The functionality of the prepared copolymer, ceramic materials, and the composite was investigated through FTIR spectroscopy. The Figure 1A(a–f) corresponds to the FTIR spectra of prepared composites. Figure 1A(a) reveals that the FTIR spectrum of prepared selenium nanoparticles via the sodium alginate templated method. Figure 1A represents the peaks of triply degenerated asymmetric stretching vibration of hydroxyl (γ_3_) mode of phosphate groups were found at 1,096 and 1,042 cm^−1^. The peak at 1,016 cm^−1^ corresponds to a non-degenerate symmetric stretching vibration (γ_1_) of the phosphate groups. The peaks found at 608 and 563 cm^−1^ resulted from the doubly degenerate bending mode (γ_4_) of the P-O bond. Hence, all the peaks seen in this spectrum robustly validate the configuration of HAP (Sumathra et al., 2018). The phosphate group's stretching frequency also revealed the existence of TCP at 870 cm^−1^ [Figure 1A(b)]. Selenium doped substituted Hap composite spectrum is given in Figure 1A(c). Based on the reference spectrum of HAP, the occurrence of HA-specific bands was retained; additionally, two new bands at 737 and 952 cm^−1^ are identified, and it due to the ${\text{SeO}}_{3}^{2-}$ ions substation HAP (Wang et al., 2012; Ma et al., 2013). The intensity increased with the content of selenium nanoparticles ions in Figure 1A(c). The LA-PEG-AS copolymer FTIR spectra were showed in Figure 1A(d). The monomer PEG, LA, AS to form copolymer ester group linkage of C-O, C=O, and C-H stretching was observed on 1,000–1,300, 1,737, and 2,890 cm^−1^, which confirms the copolymer formation. Figure 1A(e) denotes FTIR spectrum of the HAP-Se/LA-PEG-AS composite, the HAP-Se and LA-PEG-AS polymer peaks are retained in this spectum. It confirm there are no structural changes after the reinforcement of copolymer with HAP-Se and polymer. Only changes the sharp peaks of HAP-Se were changed to slightly broad due to the electrostatic interaction of polymer NH_2_ and -OH group of HAP molecule. And Figure 1A(f) corresponds to VCR loaded HAP-Se/LA-PEG-AS composite. The ester functional C=O group stretching peak gets sharpened due to the ester functional group of VCR molecule. From the materials characterization, functional group peaks are confirmed the presence of a component in the materials.

### XRD Analysis

The diffraction peaks of the Se, HAP, HAP-Se, LA-PEG-AS, HAP-Se/LA-PEG-AS, and HAP-Se/LA-PEG-AS/VCR composites are presented in the XRD pattern. The X-ray diffraction spectrum primarily authenticates the selenium nanoparticles formation in the presence of sodium alginate. The obtained XRD pattern of as-prepared Se nanoparticle is shown in Figure 1B(a). All the peaks of pure Se at 2θ = 23.231, 29.430, 43.32 degrees indexed to (100), (101), (102). The XRD pattern suggests that the Se is in nanocrystalline nature and matches very well in agreement with the literature value (JCPDS File No. 06-0362). All peaks can be indexed to the hexagonal of Se structure, with the absence of any impurities. The main peaks of HAP formed at 25.75, 31.6, 32.92, 34.29, 39.76, 46.82, and 54.10 degree could be indexed to (002), (211), (112), (300), (202), (222), and (213) lattice planes of the hexagonal HAP, respectively [Figure 1B(b)]. It demonstrated that diffraction is more reliable than the standard diffraction pattern (JCPDS card no. 09-0432). Figure 1B(c) shows the HAP-Se composite and Se retained's corresponding peaks with the crystalline nature of Se and HAP composite. Figure 1B(d) corresponds to the prepared LA-PEG-ASP polymer XRD pattern shows the significant PEG peaks at 18.90 and 23.009. Figure 1B(e) corresponds to the composite of HAP-Se/LA-PEG-AS; from the XRD pattern, the corresponding peaks of HAP-Se and LA-PEG-AS Polymer were retained. But the crystallinity composite slightly increased because PEG's presence reduces the original crystallinity nature of the HAP-Se (Jayaramudu et al., 2016). Figure 1B(f) represents the vincristine sulfate loaded HAP-Se/LA-PEG-AS composite. Besides, the crystallinity of composite gets increased after loading Vincristine sulfate due to the drug's crystalline nature. A similar pattern was observed after the loading of VCR in the HAP-Se/LA-PEG-AS composite. It means that the VCR molecules are in crystalline form, and drug crystal nature detected in the HAP-SE/LA-PEG-AS/VCR formulation (Tang et al., 2019).

### Morphological Analysis

**Figure 2:**
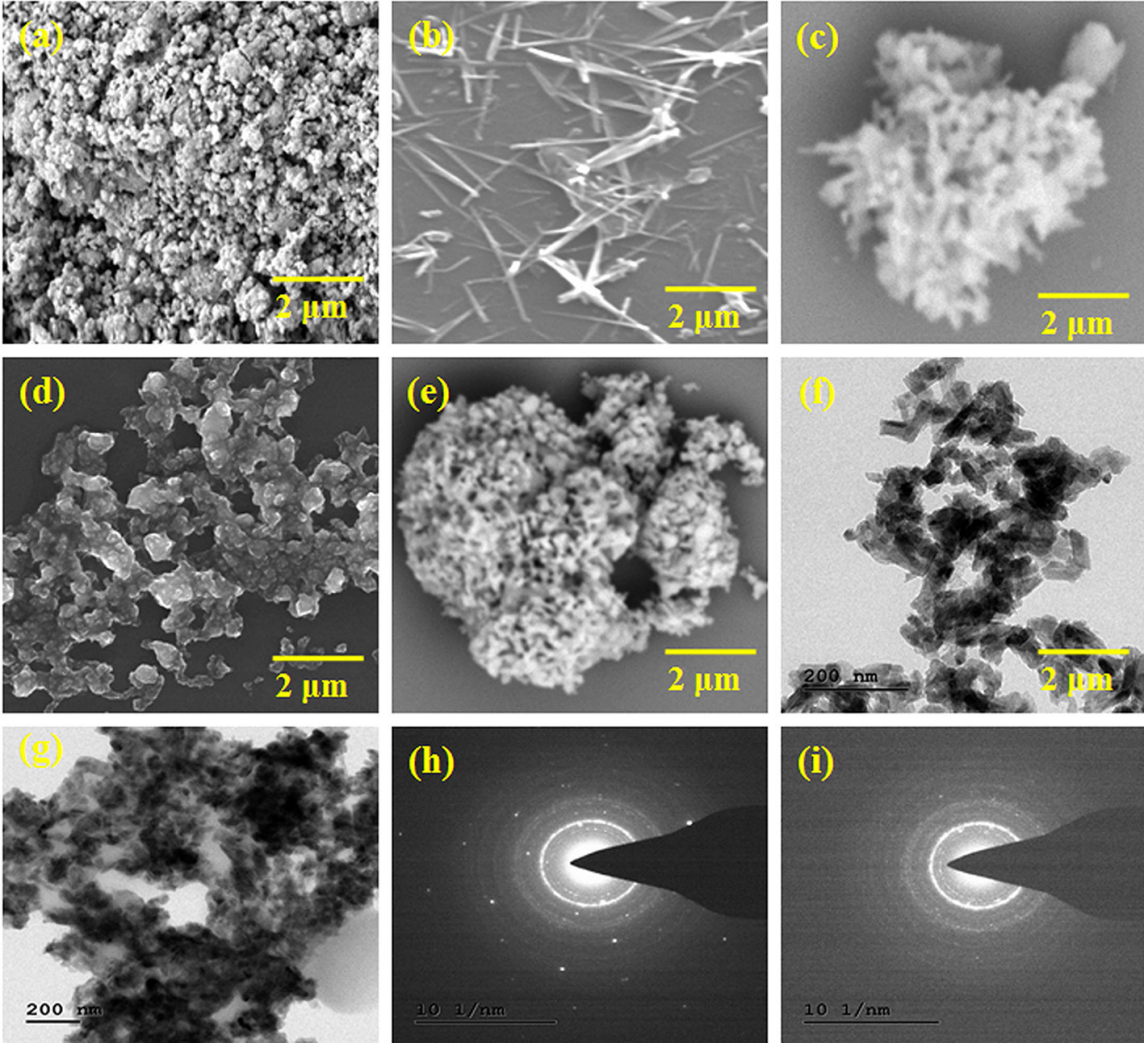
SEM images of **(a)** HAP, **(b)** Se nanoparticles, **(c)** HAP-Se, **(d)** HAP-Se/LA-PEG-AS **(e)** HAP-Se/LA-PEG-AS/VCR, TEM images, and SAED image of **(f,h)** HAP-Se/LA-PEG-AS **(g,i)** HAP-Se/LA-PEG-AS/VCR.

The morphology of the obtained HAP, Se nanoparticles, HAP-Se, HAP-Se/LA-PEG-AS, and HAP-Se/LA-PEG-AS/VCR composites observed by using the SEM technique. The experimental results for all the materials are showed in Figure 2. Figure 2a indicates that HAP particles formed in a nano-size range due to the influence of probe ultra-sonication and thermal assistance. Figures 2b–e shows the Se nanoparticles' morphology, HAP-Se, HAP-Se/LA-PEG-AS, and HAP-SE/LA-PEG-AS/VCR composites. The SEM images represented in Figure 2b indicate that the Se has a rod-like morphology. The addition of a 1% concentration of Se containing HAP resulted in a change in the morphology compare with pure HAP. The nanorods Se influence the HAP-Se morphology by the high-temperature sintering process (Sintering-600°C). Figure 2d represents the SEM image of polymer LA-PEG-AS and its aggregated spherical-like morphology. Figure 2e corresponds to the vincristine loaded polymer composite HAP-SE/LA-PEG-AS composite; it shows interconnected particles with porous nature. And it looks like an extracellular matrix (ECM) with scaffold morphology. Further investigation of the TEM instrument, the TEM images of the HAP-Se/LA-PEG-AS and HAP-SE/LA-PEG-AS/VCR composites are presented in Figures 2f,g. The morphology of the composites was well-connected with the SEM observations. Both the TEM images of the HAP-Se/LA-PEG-AS and HAP-Se/LA-PEG-AS demonstrate that the inner particles are incredibly dense, and the outer particles were less dense. Therefore, it concluded that the VCR loaded in HAP-Se/LA-PEG-AS composite material. Figures 2h,i correspond to SAED images of HAP-Se/LA-PEG-AS and HAP-Se/LA-PEG-AS, respectively, confirming the semi-crystalline nature of the prepared composites to tissue regeneration.

### Contact Angle Measurement

**Figure 3:**
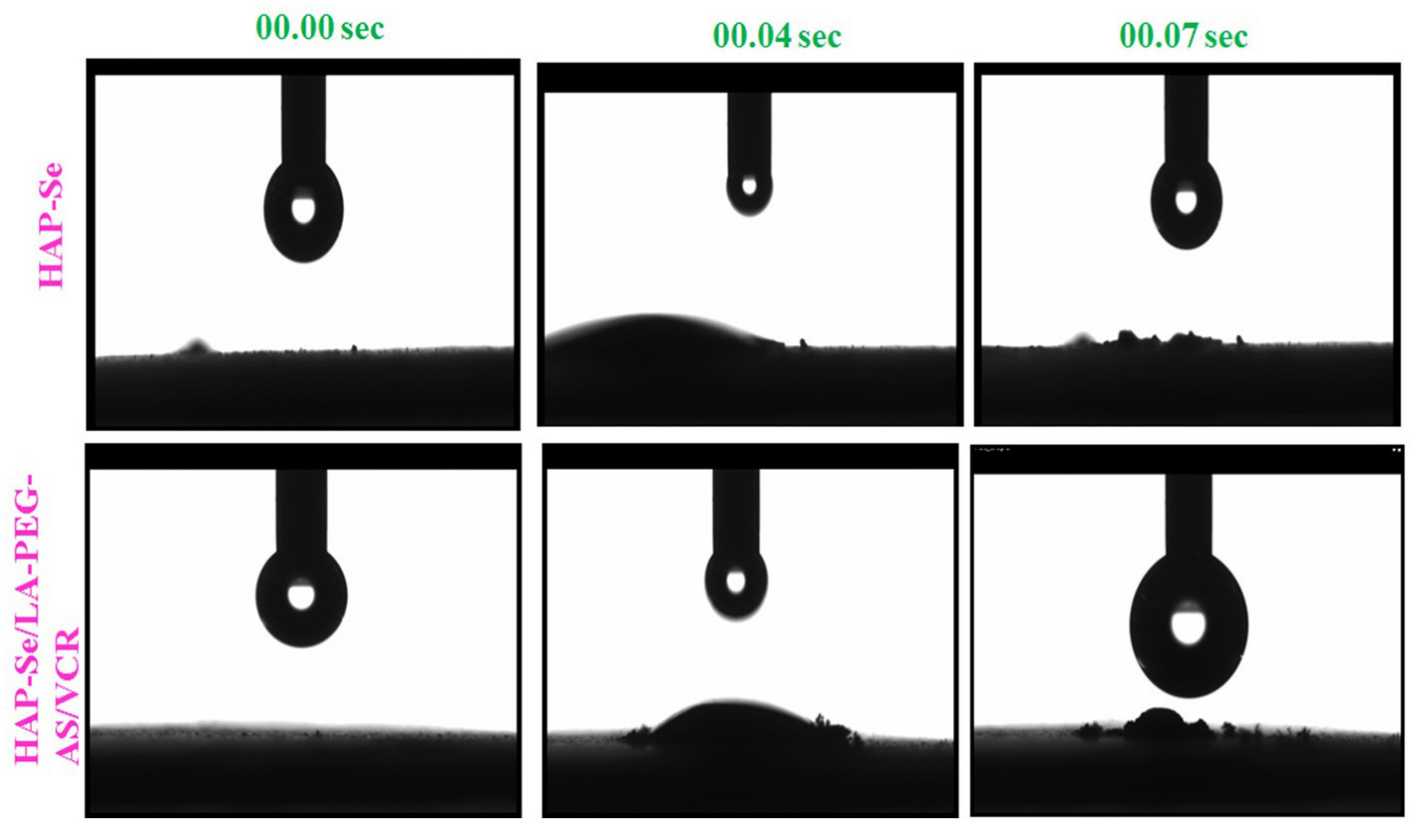
Optical images Contact angle measurements of ultrapure water droplets pipetted on specimens on HAP-Se, HAP-Se/LA-PEG-AS/VCR at 00.00, 00.04, and 00.007 S.

The surface wettability of biomaterials intensively influences the cell attachment, and the hydrophilicity of the biomaterials was accessed by measuring the water contact angle (WCA). The average water contact angle at different times of biomaterial was showed in Figure 3. From the results, HAP-Se, HAP-Se/LA-PEG-AS/VCR observed as slightly hydrophilic. Th hydrophilic nature is due to the presence of LA-PEG-AS polymer in the composite material. Lin et al. (1994) described a hydrophilic surface that enhances cell adhesion in cell culture. Expecting an endorsement effect on cell attachment and growth were introduced to improve wettability. As shown in Figure 3, the rapid water insertion process (0.04 and 0.07 S) indicates the hydrophilicity of HAP-Se/AS-PEG-LA/VCR. The droplet is firmly attached to the biomaterial, which is hydrophilic nature. The contact angle of the biomaterial may present between 0 and 30°C.

### VCR Loading Capacity and *in-vitro* VCR Release Analysis

**Figure 4:**
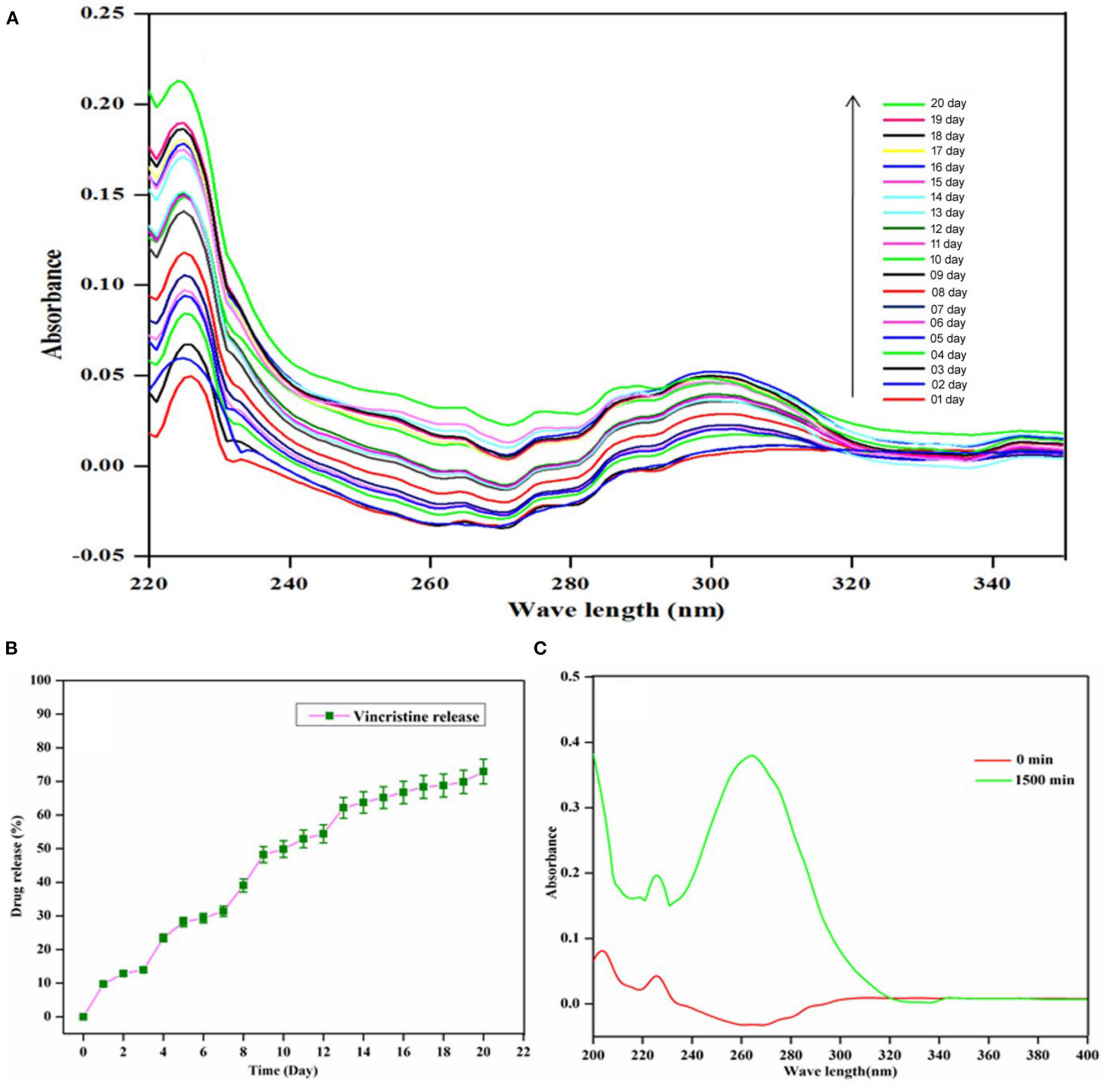
*UV-vis* Spectra of VCR **(A)** VCR release from HAP-Se/LA-PEG-AS/VCR; **(B)** Cumulative release profile of VCR from HAP-Se/LA-PEG-AS/VCR; **(C)** Loading capacity of HAP-Se/LA-PEG-AS.

The plan of the investigation is the self-repairing implant development for osteosarcoma treatment through anticancer drug-loaded composite. The UV-Visible spectroscopy analysis observed the examination of the loading capacity of the HAP-Se/LA-PEG-AS composite and its releasing abilities, and the results are given in Figures 4A–C. Figure 4C indicates UV-visible spectra of the VCR concentration at zero min and 120 min after vertexing the HAP-Se/LA-PEG-AS/VCR composite. Initially, the absorption peak of VCR appearing at the intensity range of nearly zero, then for the composites of the vortex for 3 h, the intensity increased by almost 0.39. The VCR loading capacity of HAP-Se/LA-PEG-AS composite was observed around 78.0%. The *in-vitro* UV-visible spectra of VCR release from HAP-Se/LA-PEG-AS/VCR was performed in the medium of PBS at pH 7.4, along with the related expulsion statement depicted in Figure 4A. The VCR release was 72.93%, over 20 days for the composites HAP-Se/LA-PEG-AS/VCR. It could be understood that from the VCR releasing profile of HAP-Se/LA-PEG-AS/VCR established, the requisite amount of drug release was observed with a constant releasing rate. The controlled release could be partially due to pores like morphology with excellent holding capacity and the length of drug releases from the HAP-Se/LA-PEG-AS/VCR composites. The steady release rate also affirms that the composite can be a potential scaffold for curing osteosarcoma diseases and helps new bone formation (Vallet Regi and Fernandez, 2011). Figure 4B represents VC's cumulative release from the HAP-Se/LA-PEG-AS/VCR composite.

### MIC Determinations

**Figure 5:**
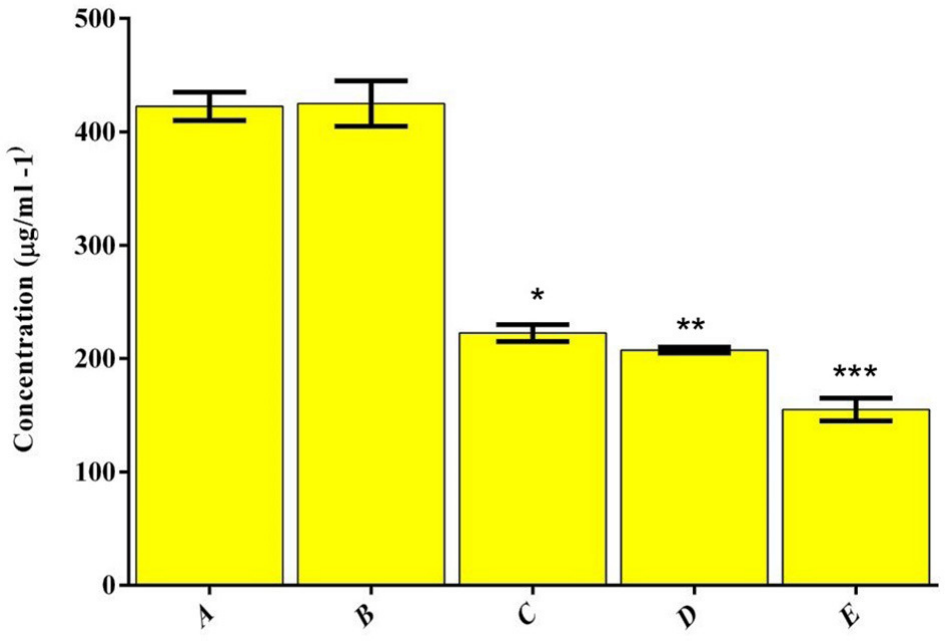
Distribution of MIC values for the polymer and drug combinations tested against *MRSA*.(A) HAP, (B) LA-PEG-AS, (C) Se, (D) HAP-Se, (E) HAP-Se/LA-PEG-AS/VCR. *, **, *** is the significance value as compared to the control (*P* ≤ 0.05).

Evaluate the antibacterial action of the prepared (A) HAP, (B) LA-PEG-AS, (C) Se, (D) HAP-Se, (E) HAP-Se/LA-PEG-AS/VCR was performed in the broth dilution assay. Their respective MIC values are represented in Figure 5. The MIC esteems acquired for MRSA were resolved in TSB fluid cultures and expressed in Figure 5. Following 24 h of hatching under oxygen-consuming conditions, variety in the turbidity degree from clear to overcast saw for all the wells containing composites showing the microbes' development hindrance. As shown, the MIC concentration of HAP-Se/LA-PEG-AS/VCR was lower than those obtained for other HAP, and LA-PEG-AS composite. When comparing HAP with Se, HAP-Se, HAP-Se/LA-PEG-AS, and HAP-Se/LA-PEG-AS/VCR were improved growth inhibition efficacy (MIC- 407 ± 21 μg/ml). The bacterial growth inhibition for HAP-Se/LA-PEG-AS/VCR composite was observed at a concentration as low as 145 ± 13 μg/ml. The variation in the expansion and drop in OD_600_ was found in the positive control wells, which contain drug alone (Se), indicating the retardation of microbial growth. In contrast, the negative control group and blank control showed prominent OD_600_ and turbidity.

### Assessment of Biofilm Biomass and Film Imaging

**Figure 6:**
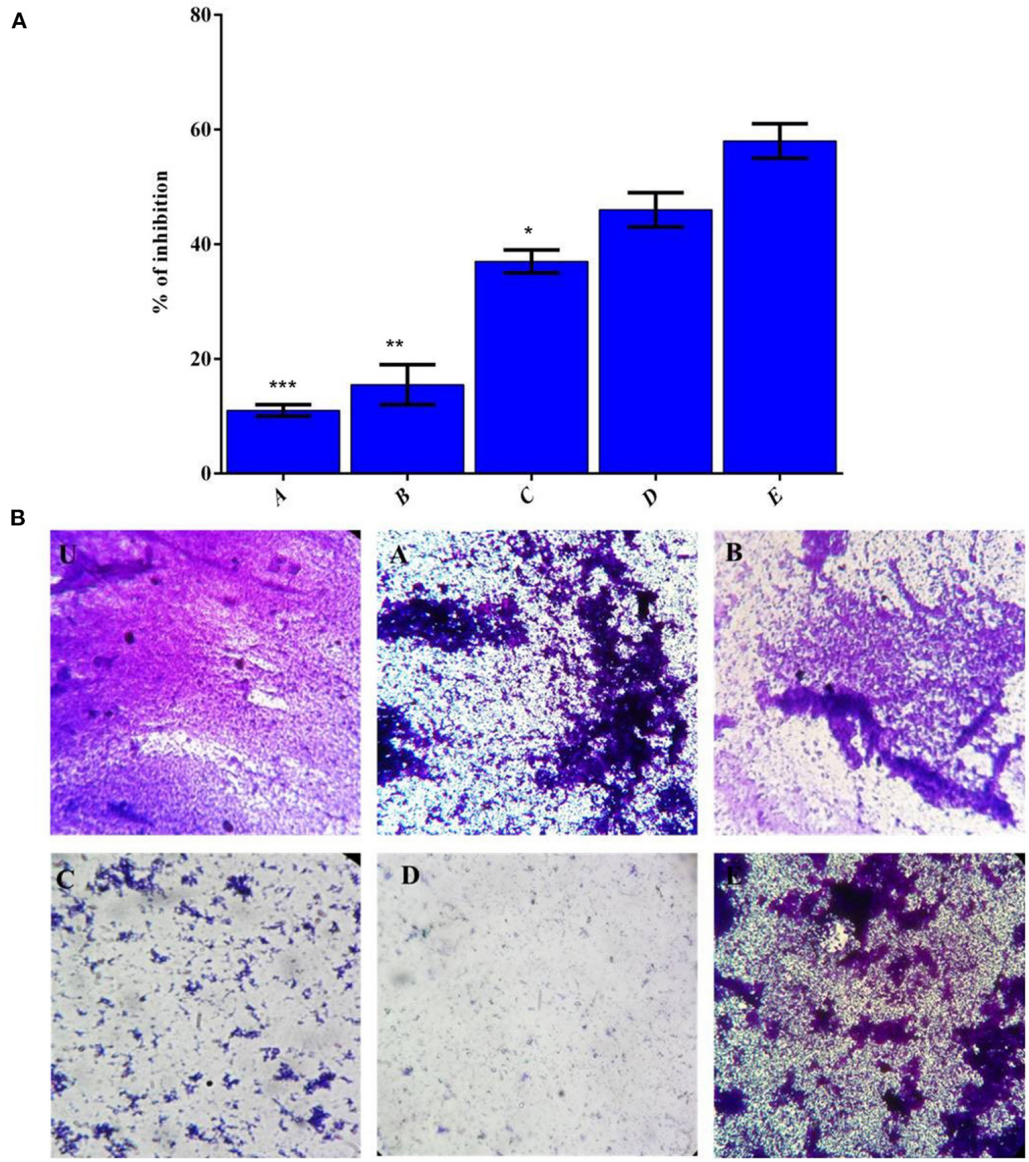
**(A)** The effect of polymer complex on biofilm formation of *MRSA* strains as measured by CV staining and determining optical density at 570 nm. **(B)** CV stained image (U) Control, (A) HAP, (B) LA-PEG-AS, (C) Se, (D) HAP-Se & (E) HAP-Se/LA-PEG-AS/VCR, Mean values of triplicate independent experiments, and SD shown. *, **, *** is the significance value as compared to the control (*P* ≤ 0.05).

The counter biofilm adequacy of HAP based polymer composite was assessed under *in vitro* condition by deciding the CV's official to the disciple biofilm of *MRSA* on 48-well MTP and appeared in Figure 6. The biofilm imaging examination results were inconsistent with the biofilm inhibition in a CV quantification assay (Figure 6A). Thus, after envisioning the biofilm after 16 h of hatching, the control CV wells demonstrated very much shaped thick biofilm progress of MRSA (Figure 6B). The MRSA indicated the demolition of biofilm development on treatment with the composite, selenium, and VCR drug mixes in actuality. *MRSA* can follow and shape biofilms outside of food handling hardware made of polystyrene, polymers, plastic, glass, Teflon, elastic, and hardened steel (Lundén et al., 2000; Chavant et al., 2002; Borucki et al., 2003; Ilan, 2003; Møretrø and Langsrud, 2004).

### Prevention of Hemolysis

**Figure 7:**
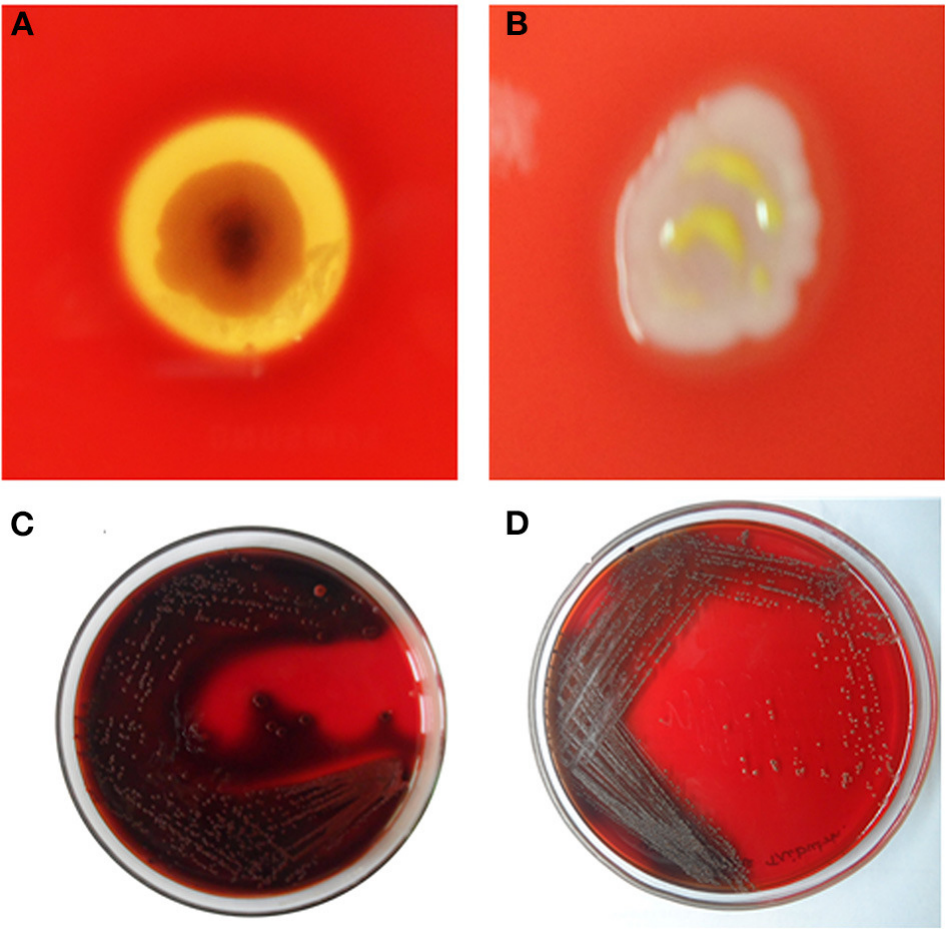
Inhibition of Haemolysin by **(A)**- Untreated control with clear RBS lysis on HAP-Se/LA-PEG-AS/VCR and **(B)** MRSA cells exposed to ½ MIC of HAP-Se/LA-PEG-AS/VCR; Inhibition of slime synthesis by **(C)** Untreated control with Congo red adsorption on HAP-Se/LA-PEG-AS/VCR and **(D)** MRSA cells exposed to ½ MIC of HAP-Se/LA-PEG-AS/VCR.

After the incubation with the 1/2 MIC of composites, the percentage of hemolysis decreased up to 62 ± 2% (HAP-Se/LA-PEG-AS/VCR), 26 ± 4% (HAP-Se), 15% (Se) 7% (LA-PEG-AS), 5% (HAP) for MRSA, respectively. The HAP-Se-LA-PEG-AS/VCR inhibits MRSA induced hemolysis of erythrocytes was significantly inhibited and shown in Figures 7A,B.

### Inhibition of Slime Production With Congo Red Agar (CRA)

Sludge recognition utilizing Congo red plates customarily used to identify biofilm-framing staphylococci, and consistent with the microscopic biofilm results, slime production by MRSA was markedly reduced by HAP-Se/LA-PEG-AS/VCR at 75 μg/mL. Noticeably, the HAP-Se/LA-PEG-AS/VCR treated MRSA cells produced the least slime, whereas the control cells made large amounts (Figures 7C,D).

### Cell Viability Against L929 Cells

**Figure 8:**
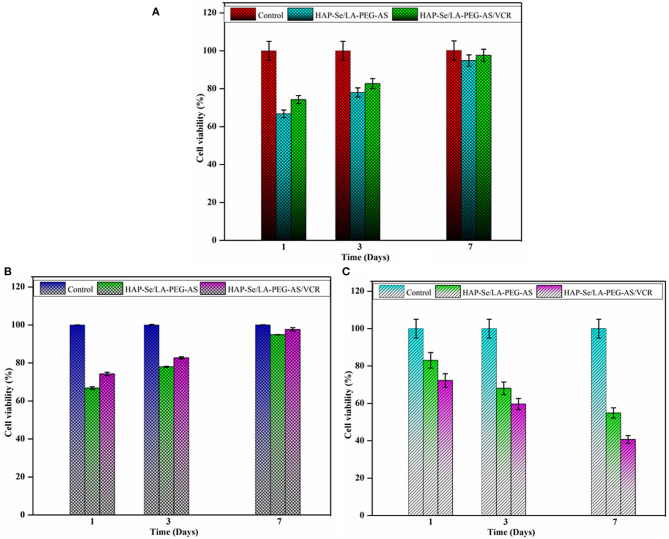
**(A)** Cell viability of L929 cell line on HAP-Se/LA-PEG-AS and HAP-Se/LA-PEG-AS/VCR at 1, 3, and 7 days; **(B)** Cell viability of MG63 osteoblasts cells on HAP-Se/LA-PEG-AS, HAP-Se/LA-PEG-AS/VCR, and control as MG63 osteoblasts like cells; **(C)** Cytotoxicity of Saos-2 Osteosarcoma cells on HAP-Se/LA-PEG-AS, HAP/LA-PEG-AS/VCR, and control as Saos-2 cells Osteosarcoma like the cell (*n* = 3, *p* < 0.005).

The biocompatibility of the prepared HAP/LA-PEG-AS and HAP/LA-PEG-AS/VCR composites tested against fibroblast (L929) cells. Cells adhered and began spreading observed was investigated from 1, 3, and 7 days on HAP/LA-PEG-AS and HAP/LA-PEG-AS/VCR composites. The HAP/LA-PEG-AS/VCR shows more proliferation than HAP/LA-PEG-AS (Figure 8A). The high cell adhesiveness of HAP/LA-PEG-AS/VCR was indicating a VCR role with higher viability on the L929 cells, and it was observed 97.68% cell viability in 7 days seeding (Figure 8A). The results conclude that the naturally dynamic polymers, for example, PEG to the hydroxyapatite, will improve the cell reactions and adhesion with the fibroblast cells. The PEG improves dispersion, mechanical, and crystallinity when it binds with lactic acid and aspartic acid. The copolymer has biodegradable, biocompatible, non-antigenic, non-toxic, organically glue, antimicrobial properties. What's more, the PEG will improve expansion and cell relocation (Jayaramudu et al., 2016). The high compatibility and excellent cell growth of HAP/LA-PEG-AS/VCR indicate the VCR loaded composites have prospective usability for bone tissue engineering.

### Cell Viability Against MG63 Cells

The biocompatibility and bioactivity of biomaterial utilized for bone tissue designing applications have practically assessed with MG63 osteoblasts like cells with the synthesized HAP/LA-PEG-AS, HAP/LA-PEG-AS/VCR composites, and MG63 osteoblasts like cells used as a control. The outcomes that the cell practicality gets expanded with increasing the days, which is appeared in Figure 8B. The composite is containing every particle group, the cell developing segments from itself. The PEG polymer enhances cell development and gives a supplement to the cells. Lactic acid and aspartic acid could also assume a significant job in a bone fix and tissue regeneration. It is more conducive to play a supporting role in polymer composites. Figure 8B shows that the addition of VCR to the composite significantly improved the biological properties at 7 days up to 97.68%. Since the vincristine loaded polymer composite shows, more viability compares with the HAP/LA-PEG-AS composite. Since the HAP/LA-PEG-AS/VCR polymer composite can be used as a biomaterial to coat on the surface-treated titanium plate to implant bone regeneration/repair.

### Cytotoxicity Against Saos-2 Cells

The cytotoxicity of HAP/LA-PEG-AS and HAP/LA-PEG-AS/VCR samples in Saos-2 cells was investigated, and the toxicity profile of the HAP/LA-PEG-AS/VCR composite was depicted in Figure 8C. Critical contrasts in the toxicity of composite were seen in Saos-2 cells after 7 days incubation and credited to the presence of VCR in the composites. This outcome exhibited that the HAP/LA-PEG-AS/VCR increased the extension of destructive cells following 7 days of incubation. The MTT measure shows that the cell reasonability diminished by 25%, in the incubation of Saos-2 cells with HAP/LA-PEG-AS/VCR composites inhibited the cell development following 7 days. Along these lines, the HAP/LA-PEG-AS/VCR composite upgrades Osteoblast action and reduces the Osteosarcoma movement. On account of the natural attribute of Vincristine Sulfate, incorporate cancer prevention agent mitigating and anticancer properties. Vincristine Sulfate blocks cell proliferation induces apoptosis in tumor cells. The cytotoxicity percentage increases with the increase of days. The results of the cytotoxicity of the composite against Saos-2 is evidence for the application of materials used in osteosarcoma treatment.

### Histopathology Analysis

**Figure 9:**
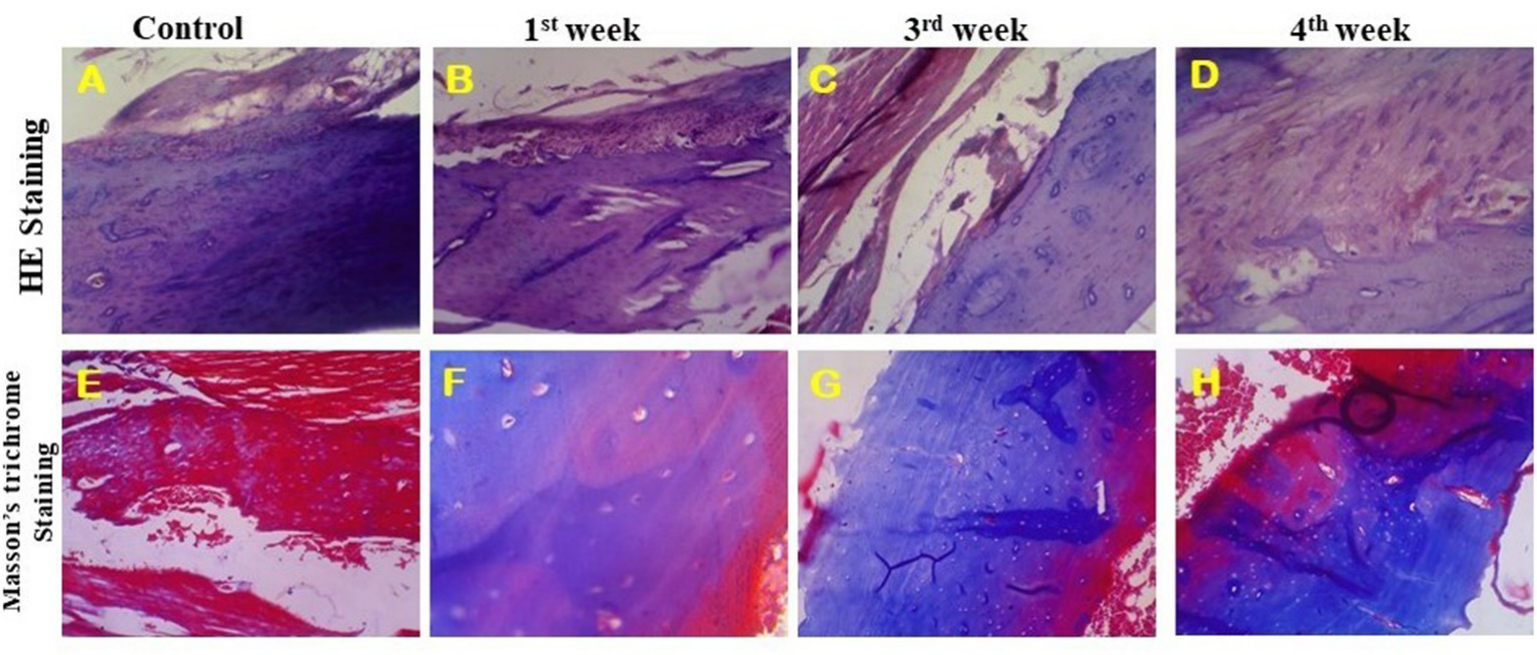
HE stained images of implanted tibia tissue at 40X **(A–D)** and Masson's trichrome stained images of implanted tibia tissue at 40X **(E–H)**.

The HAP/LA-PEG-AS/VCR composite coated Ti plate was investigated in a rat animal model, and the defected bone, the regeneration ability of the artificial implant, was characterized by histopathological analysis. Figure 9 represents a histopathological observation of HAP-Se/LA-PEG-AS/VCR composite coated Ti plate implanted in Wistar rats. Figures 9A–D illustrates the HE stained tissue images of control, 1st, 3rd, and 4th week of post-implantation, respectively. The HAP-Se/LA-PEG-AS/VCR composite results in new bone tissue generation by hematopoietic cells, new bone cells, and tibia bone. From Figures 9A–D, the blue color disappearance indicates the bone cell gets matured and form the new bone by the implant compared with the control result. Figures 9E–H corresponds to the Masson's Trichrome stained (MTS) tissue images of control, 1st, 3rd, and 4th week of post-implantation of Wister rats, respectively. Masson's Trichrome stained bone tissue at 4 weeks, results from new bone development. It confirms HAP/LA-PEG-AS/VCR composite coated titanium plate implanted on the defect site with the existence of osteoblasts and osteocytes beside with collagen and bone (Figures 9F,G). At 4 weeks, the matured bone surrounding with dense collagen and cartilage, and traces of HAP/LA-PEG-AS/VCR were manifest in the defect site (Figure 9H).

### Radiograph Analysis

**Figure 10:**
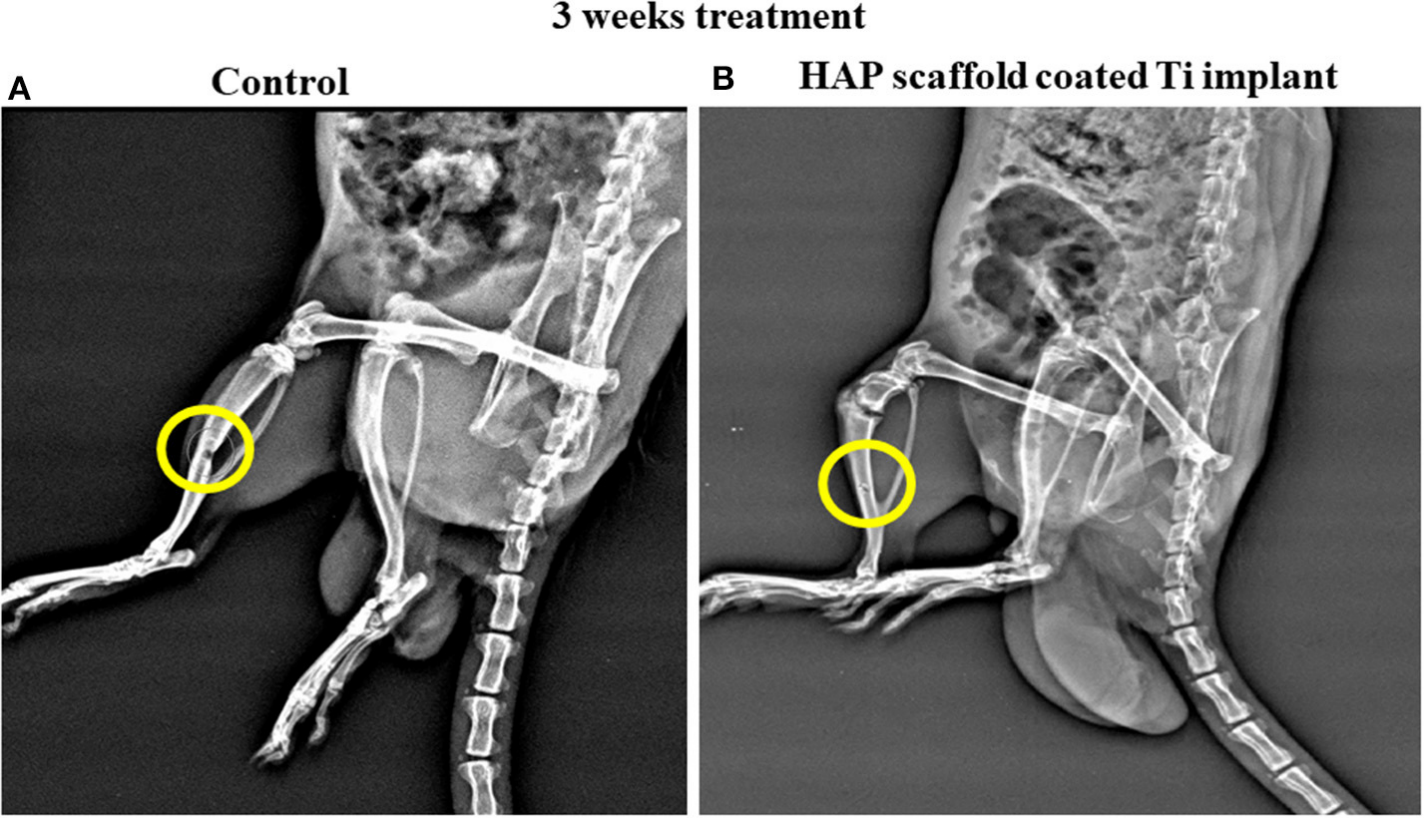
The X-Ray radiography of control and HAP/LA-PEG-AS/VCR composite coated Titanium implant at 3rd weeks of post-operative in rat animal model.

As appeared in the zone under the X-Ray picture's spotted circle in Figure 10A, the primarily measured tibia bone deformity was made by mechanical drilling. HAP-Se/LA-PEG-AS/VCR coated titanium plate filled in the defect created rat. The HAP-Se/LA-PEG-AS/VCR composite coated Ti plate could well change the lopsided bone defect to the new bone formation by permitting osteoblast cells' multiplication Ti plate embedded in the 3rd week of investigation. The results are presented in Figure 10B, where it was observed the new bone was regenerated into a defective place. From these outcomes, the HAP-Se/LA-PEG-AS/VCR composite could positively affect the tibia bone deformity. The X-ray radiography results could relate to the histopathological results.

## Discussion

The prepared selenium's functionality, ceramic materials, polymer, and the polymeric ceramic composite were investigated through FTIR spectroscopy. The Figure 1A(a-f) corresponds to the FTIR spectra of prepared (a) Se-NPS, (b) HAP, (c) HAP-Se, (d) LA-PEG-AS, (e) HAP-Se/LA-PEG-AS, (f) HAP-SE/LA-PEG-AS/VCR composites. The ester C=O stretching peak gets sharpened from the FTIR spectrum due to the ester functional group of the VCR molecule. The X-ray diffraction spectrum primarily authenticates selenium nanoparticles' formation in the presence of sodium alginate (Yanhua et al., 2016; Zhou et al., 2020). The diffractogram of HAP-Se/LA-PEG-AS composite was observed a similar pattern of VCR loaded HAP-Se/LA-PEG-AS, and no diffraction changes were observed after the VCR loading in HAP-Se/LA-PEG-AS composite (Tang et al., 2019). It is due to the VCR molecules are in crystalline form, and the drug crystal nature not affected the crystalline nature of HAP-SE/LA-PEG-AS. Morgan et al. (1996) revealed that lower hydroxyapatite crystallinity brings about a higher ECM mineralization measure. As a component of embedding, the material's crystallinity is outcomes, recommend that Ca-P dissolution create organic mineralization. Nonetheless, it is unclear whether substrates with low crystallinity and a layer of exceptionally mineralized *in vitro* shaped bone-like tissue will start the arrangement of coordinated bone *in-vivo*. It is imprecise if this is more profitable contrasted with a substrate with a higher crystallinity and a layer of refined cells with a delivering network that isn't or just ineffectively mineralized.

The surface morphology of the prepared HAP, Se nanoparticles, HAP-Se, HAP-Se/LA-PEG-AS, and HAP-Se/LA-PEG-AS/VCR composites observed and indicate rod-like, aggregated spherical-like, and a rod inserted spherical morphology. Natural bone combines organic and inorganic composite containing all around organized collagen fibrils, nanocrystalline, and rod-like inorganic material with a length of 25–50 nm. The bone structure arrangement is shaped from seven degrees of order and mirrors every segment's material and mechanical properties (Jeevanandam et al., 2018). The primary issue of developing bone scaffolds is evolving a biomaterial with functions similar and characteristics to natural bone. The factors associated with scaffold properties, such as mechanical properties, porosity, biocompatibility, surface, and biodegradability, are also significant for developing artificial bone scaffolds. Here the developed HAP-SE/LA-PEG-AS biomaterials are overcome to issues, and the results are better correlated with the natural mimicking bone materials (Colosi et al., 2015).

Th hydrophilic nature is due to the presence of LA-PEG-AS polymer in the composite material. As Murugan et al. (2018) depicted, a hydrophilic surface improves cell bonds in cell culture. Expecting an advancement impact on cell connection and development was acquainted with improve wettability. The investigation aims to investigate the self-repairing of bone cancer treatment by the loading anticancer drug-loaded composite. The examination of VCR loading capacity and releasing properties of the HAP-Se/LA-PEG-AS/VCR composite material was investigated. The results indicate HAP-Se/LA-PEG-AS composite having 78.0% of VCR loading capacity, and the VCR released sustainably up to days as 72.93% from the loaded VCR drug.

The biomaterial research is currently subjected to improving implanted device significance for fast bone regeneration to disease affected tissues and reducing the implanted site's further side effect after post-implantation. The bacterial growth inhibition for HAP-Se/LA-PEG-AS/VCR composite was observed at a concentration as low as 145 ± 13 μg/ml. The adjustment in the development and drop in OD_600_ was found in the positive control wells, which contain Se alone, demonstrating the hindrance of microbial development. Interestingly, the negative control group and clear control indicated raised OD_600_ and turbidity. Noticeably, the HAP-Se/LA-PEG-AS/VCR treated MRSA cells produced the least slime, whereas the control cells made large amounts (Figures 7C,D). Biocompatibility of the HAP/LA-PEG-AS and HAP/LA-PEG-AS/VCR composites tested against fibroblast (L929) cells, and it observed good viable nature. The chitosan has biodegradable, biocompatible, non-antigenic, non-toxic, biologically adhesive, antimicrobial properties. What's more, the gelatin will improve multiplication and cell relocation due to its hydrophobic nature (Mota et al., 2014). The high similarity and fantastic cell development of HAP/LA-PEG-AS/VCR show the VCR stacked composites have planned convenience for bone tissue designing.

The present *in-vivo* results were allied with the previous investigation by the various researchers. Recently, Prabakaran et al. (2020) investigated the polydopamine-treated Ti plant coated with lanthanides substituted hydroxyapatite with Aloe vera gel. The researchers have observed the regeneration ability of the implant 4th week in the rat model. Similarly, the titanium fibers observed the regeneration ability, and it is prepared by the compression and shear stress at normal room temperature (Takizawa et al., 2018). Gelatin methacrylate/nano fish bone hybrid hydrogel was investigated the biomimetic bone regenerations. The investigation results show the materials having the potential ability to bone regenerations (Huang et al., 2020).

## Conclusion

The Artificial organs made of the biocompatible polymer can be implanted, which overcome tissue rejection problems due to immune responses, compatibility, and minerals & bioactive compound release. The Lactic Acid-Polyethylene Glycol-Aspartic acid (LA-PEG-AS), is reported here as a non-toxic, biocompatible, and biodegradable material the combination of HAP-Se for bone regeneration. The HAP-Se/LA-PEG-AS composite with VCR drug-coated implant achieved prolonged therapeutic effects. The SEM represents the morphology of vincristine loaded polymer composite HAP-SE/LA-PEG-AS/VCR it shows and interconnected particles. And it was mimic the extracellular matrix with rod-like morphology, and it correlated with TEM results. The *in-vitro* analysis of cell viability and cell cytotoxicity on HAP-Se/LA-PEG-AS/VCR composites has a good viable nature in L929 and MG63 cells. The *in-vivo* animal investigation reveals that the HAP-Se/LA-PEG-AS/VCR composites coated Titanium plate having the ability for new bone formation. The HAP-Se/LA-PEG-AS/VCR composites may be suitable for the bone implantation for repairing defected bone after clinical evaluation.

## Data Availability Statement

The datasets presented in this study can be found in online repositories. The names of the repository/repositories and accession number(s) can be found in the article/supplementary material.

## Ethics Statement

The animal study was reviewed and approved by this research was approved by the animal ethical committee of the second affiliated hospital (Tangdu Hospital), Air Force Military Medical University Approved No. SCXK (army) 2019-214.

## Author Contributions

WD and XD: experimental works and manuscript writing. ON and SA: characterization and validations. JZ and WL: design, supervision, and proofreading. All authors contributed to the article and approved the submitted version.

## Conflict of Interest

The authors declare that the research was conducted in the absence of any commercial or financial relationships that could be construed as a potential conflict of interest.
